# Unraveling Alzheimer’s disease: insights from single-cell sequencing and spatial transcriptomic

**DOI:** 10.3389/fneur.2024.1515981

**Published:** 2024-12-17

**Authors:** Yibo He, Wenqiang Lu, Xiao Zhou, Jie Mu, Wei Shen

**Affiliations:** ^1^The First Affiliated Hospital of Zhejiang Chinese Medical University (Zhejiang Provincial Hospital of Chinese Medicine), Hangzhou, Zhejiang, China; ^2^Department of Thoracic Surgery, Suzhou Kowloon Hospital, Shanghai Jiao Tong University School of Medicine, Suzhou, Jiangsu, China; ^3^Department of Pharmacy, Shuangqiao Economic and Technological Development Zone People's Hospital, Chongqing, China

**Keywords:** Alzheimer’s disease, cognitive decline, single-cell sequencing, spatial transcriptomics, neuroinflammation, microglia, astrocytes, gene expression

## Abstract

Alzheimer’s disease (AD) is a neurodegenerative disorder marked by cognitive decline, primarily affecting memory and executive function. This review highlights recent advancements in single-cell sequencing and spatial transcriptomics, which provide detailed insights into the cellular heterogeneity and neuroimmune mechanisms of AD. The review addresses the need for understanding complex cellular interactions to identify novel therapeutic targets and biomarkers. Single-cell sequencing has revolutionized our understanding by mapping gene expression at the individual cell level, revealing distinct microglial and astrocytic states that contribute to neuroinflammation and neurodegeneration. These technologies have uncovered disease-associated microglial subpopulations and gene expression changes linked to AD risk genes, essential for developing targeted therapies. In conclusion, the integration of single-cell and spatial transcriptomics with other omics data is crucial for a comprehensive understanding of AD, paving the way for personalized medicine. Continued interdisciplinary collaboration will be vital in translating these findings into effective treatments, improving patient outcomes.

## 1 Introduction

Alzheimer’s disease (AD) is the most common form of dementia, affecting millions worldwide, with prevalence rates increasing with age (1–3). The disease significantly impacts cognitive functions, particularly memory, executive function, and visuospatial skills, leading to a progressive decline in the ability to perform daily activities (4, 5). The progression of AD can be categorized into three stages: preclinical, mild cognitive impairment (MCI), and Alzheimer’s dementia, each with varying degrees of cognitive and functional impairment (1, 6). The pathological hallmarks of AD include amyloid plaques, neurofibrillary tangles, and significant neuronal loss (7). Amyloid plaques are extracellular deposits of amyloid-beta peptides, while neurofibrillary tangles consist of hyperphosphorylated tau proteins within neurons, disrupting normal cell function (8, 9). These pathologies contribute to synaptic dysfunction, neuroinflammation, and ultimately neuronal death, which are critical drivers of cognitive decline observed in AD patients (10, 11).

Single-cell sequencing is a cutting-edge technology that allows for the analysis of gene expression at the individual cell level, providing a detailed map of cellular diversity and function (4). This technique involves isolating single cells, amplifying their genetic material, and sequencing it to identify unique gene expression profiles (9). The ability to dissect the transcriptomes of individual cells provides unprecedented resolution in understanding cellular heterogeneity in complex tissues like the brain (8). In neuroimmune research, single-cell sequencing has become invaluable for identifying distinct cell types, states, and interactions within the brain’s immune landscape (12). This technology enables the detailed characterization of microglia, astrocytes, and other immune cells, revealing their roles in neuroinflammation and neurodegeneration (13). By mapping the cellular and molecular changes associated with AD, researchers can uncover new targets for therapeutic intervention and develop strategies to modulate neuroimmune responses (14).

In recent years, single-cell sequencing and spatial transcriptomics have significantly advanced our understanding of Alzheimer’s disease (AD). Studies have identified new disease-associated microglial populations and other non-neuronal cell types that play crucial roles in neuroinflammation and neurodegeneration in AD. For example, recent research has uncovered distinct microglial subpopulations with differential roles in amyloid-beta clearance and neuroinflammation (15, 16). Additionally, recent meta-analyses and systematic reviews have highlighted the potential of these technologies in identifying new biomarkers for AD (17, 18). Innovations in spatial transcriptomics, such as Visium and MERFISH, have enabled high-resolution mapping of gene expression in AD-affected brain regions, revealing how cellular interactions change in response to amyloid pathology (19). Furthermore, the integration of transcriptomics with other omic technologies, like proteomics, is providing a more comprehensive view of the molecular mechanisms driving AD, offering new opportunities for biomarker discovery and therapeutic targeting (20, 21).

## 2 Neuroimmune mechanisms in Alzheimer’s disease

### 2.1 Inflammatory responses in AD

Microglia, the resident immune cells of the central nervous system, are crucial in AD through various activation states and functions (22). Activated microglia can phagocytose amyloid-beta (Aβ) plaques, but chronic activation leads to neurotoxic substance release, exacerbating neuronal damage (23). Genetic studies have shown several AD-associated genes are predominantly expressed in microglia, suggesting their critical involvement in early disease stages (24).

Astrocytes also play a significant role in AD-related neuroinflammation. These cells become reactive and release inflammatory cytokines that can alter the neuronal environment (25). Reactive astrocytes exacerbate neuronal damage through the release of neurotoxic substances, contributing to excitotoxicity and further neuronal loss (26). Their interaction with microglia, such as the activation of P2X7 receptors by astrocytic ATP release, promotes the formation of the NLRP3 inflammasome, implicated in AD (27).

**Figure 1:**
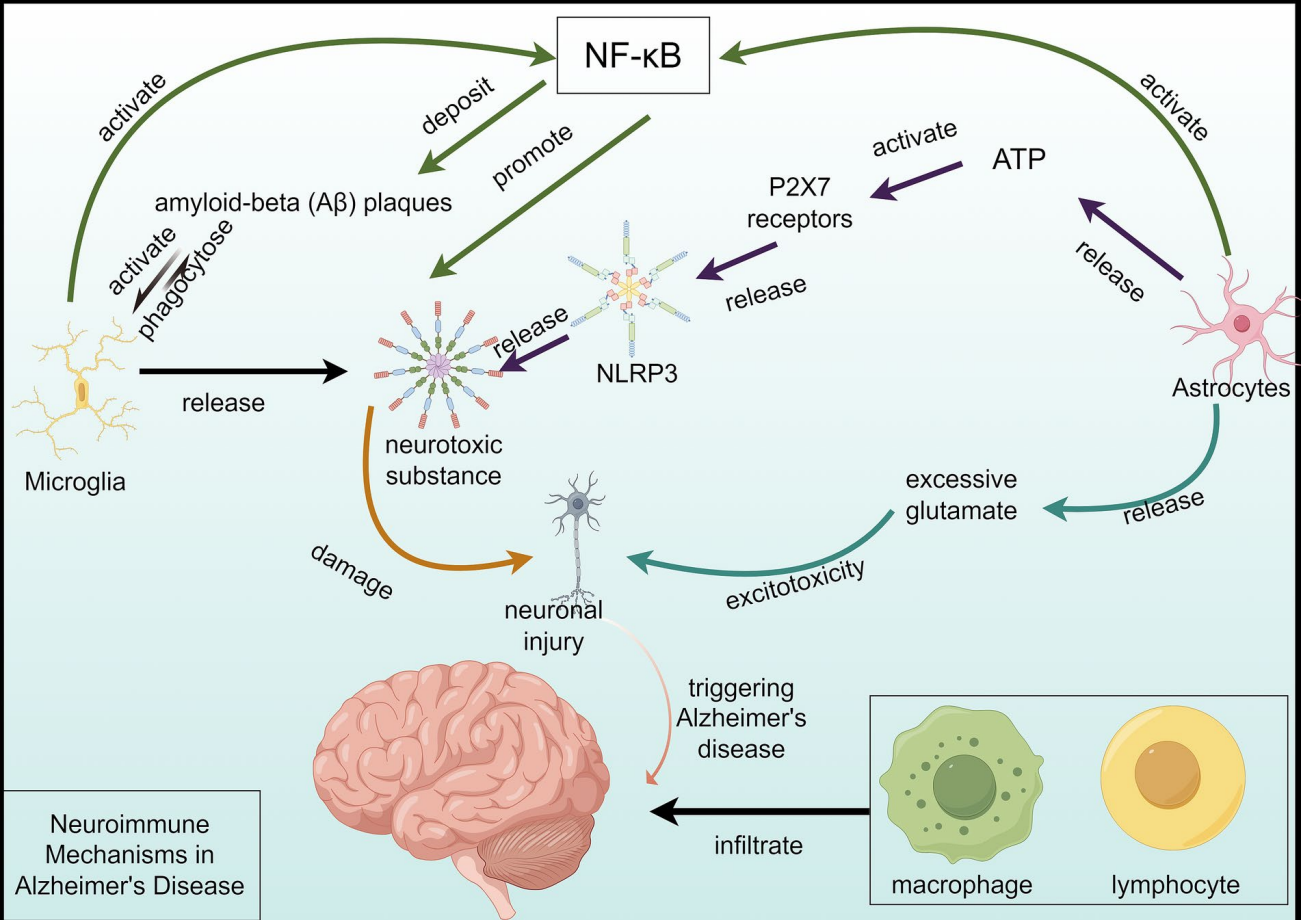
Neuroimmune mechanisms in Alzheimer’s disease. This figure depicts the complex neuroimmune mechanism of Alzheimer’s disease (AD). Microglial cells are activated by the deposition of a large amount of Aβ and engulf Aβ plaques, but the activation process releases neurotoxic substances, damaging neurons. On one hand, astrocytes can activate the formation of NLRP3 inflammasomes by releasing ATP to activate the P2X7 receptor, further releasing neurotoxic substances, exacerbating neuronal damage. On the other hand, astrocytes’ excessive release of glutamate and other excitatory amino acids can generate excitotoxicity, damaging neurons. The activation of the NF-κB pathway leads to the transcription of pro-inflammatory cytokines and Aβ deposition, resulting in neuronal damage and death. Furthermore, in peripheral immunity, the infiltration of macrophages and lymphocytes into the brain further exacerbates neural damage. Multiple factors collectively contribute to the progression of AD.

The NF-κB and NLRP3 inflammasome pathways are key mediators of the inflammatory response in AD. NF-κB activation in microglia and astrocytes leads to the transcription of pro-inflammatory cytokines, contributing to chronic neuroinflammation (27). The NLRP3 inflammasome further amplifies this response by promoting the release of interleukin-1β (IL-1β), a potent pro-inflammatory cytokine (28, 29). Targeting these pathways has shown promise in reducing neuroinflammation and improving cognitive function in animal models of AD (30) (Figure 1).

### 2.2 Immune cell heterogeneity

The brain’s immune landscape includes microglia, astrocytes, and infiltrating peripheral immune cells, each contributing uniquely to AD (31). Microglia are primary immune cells, while astrocytes participate actively in immune responses and interact closely with microglia (32). Peripheral immune cells, such as macrophages and lymphocytes, can infiltrate the brain in AD, further complicating the neuroimmune dynamics (33).

Single-cell sequencing has revolutionized our understanding of immune cell heterogeneity in the brain, identifying unique subpopulations with distinct transcriptomic signatures (30). In AD, single-cell RNA sequencing has uncovered diverse microglial states, including disease-associated microglia (DAM), characterized by upregulated genes involved in phagocytosis and lipid metabolism (34).

**Figure 2:**
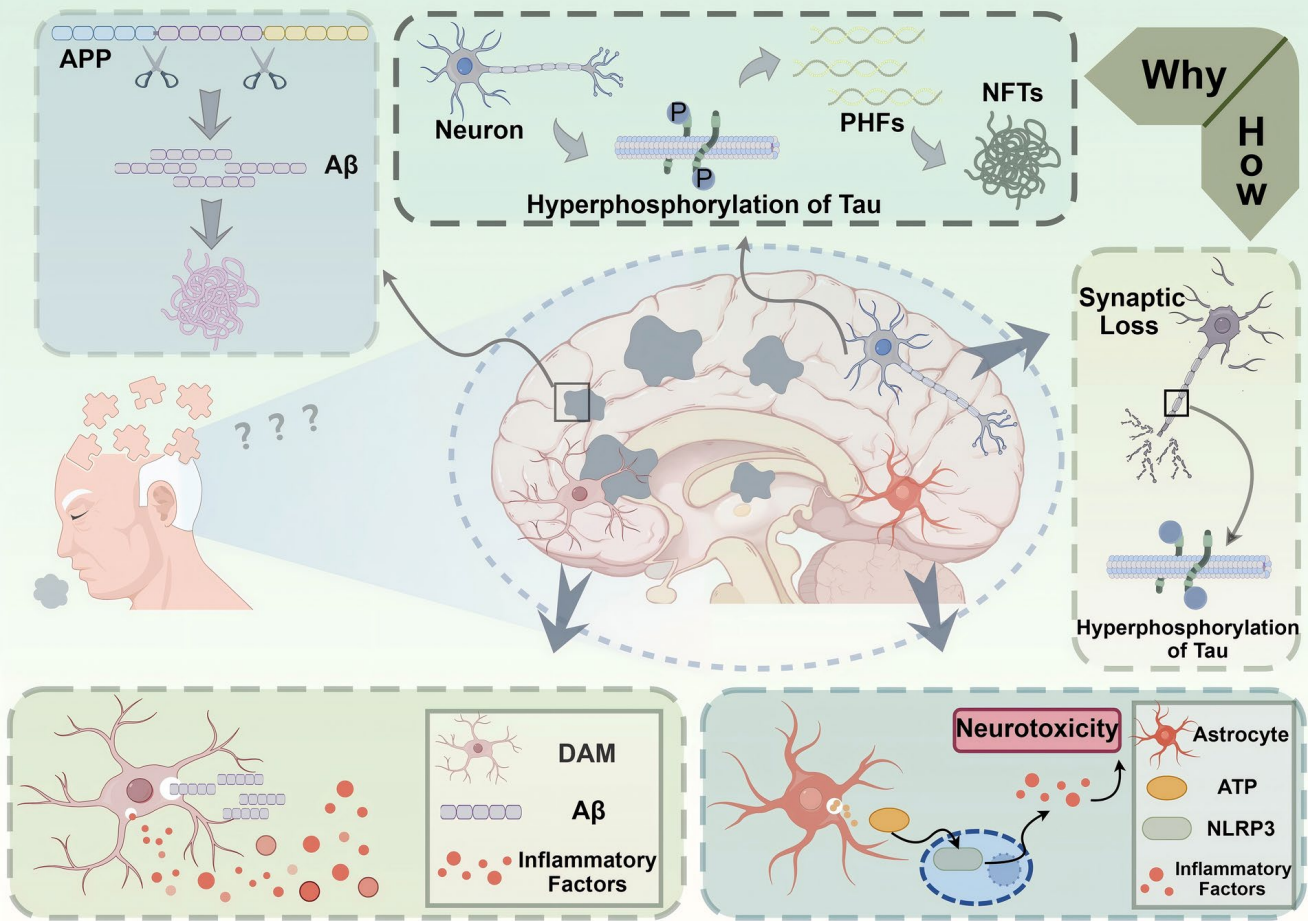
Pathological mechanisms underlying Alzheimer’s disease (AD). This figure depicts the molecular and cellular mechanisms contributing to the pathogenesis of Alzheimer’s disease (AD). The accumulation of amyloid-beta (Aβ) peptides, derived from the cleavage of amyloid precursor protein (APP), leads to the formation of extracellular Aβ plaques, a hallmark of AD pathology. Concurrently, hyperphosphorylation of tau proteins within neurons results in the formation of paired helical filaments (PHFs) and neurofibrillary tangles (NFTs), disrupting microtubule stability and intracellular transport. These processes contribute to synaptic loss and neuronal dysfunction, which are directly linked to cognitive deficits in AD. Neuroinflammation exacerbates disease progression, as shown by the activation of disease-associated microglia (DAM) and astrocytes in response to Aβ plaques. DAM release pro-inflammatory factors, further amplifying neuronal damage, while astrocytes mediate the activation of the NLRP3 inflammasome through ATP signaling, aggravating neurotoxicity. Together, these events culminate in neuronal loss and brain atrophy, manifesting as the clinical symptoms of memory impairment and cognitive decline characteristic of AD.

The functional heterogeneity of immune cells in the brain significantly impacts AD progression and pathology. Different subpopulations of microglia and astrocytes can have opposing effects on neuroinflammation, neuronal survival, and amyloid clearance, highlighting the need for targeted therapeutic approaches (15, 35). Understanding the roles of these diverse cell populations is crucial for developing effective therapies to mitigate AD pathology (31) (Figure 2).

## 3 Single-cell transcriptomics in Alzheimer’s disease

### 3.1 Role of single-cell transcriptomics

Single-cell transcriptomics (scRNA-seq) is a revolutionary technology that enables the profiling of gene expression at the single-cell level, providing unparalleled insights into cellular heterogeneity. Unlike bulk RNA sequencing, which averages gene expression across many cells, scRNA-seq captures the transcriptomic diversity of individual cells, revealing distinct cellular states and subpopulations (15, 36). This granularity is crucial for understanding complex tissues, like the brain, where different cell types and states contribute to overall function and disease progression (37). The technique involves isolating single cells, reverse-transcribing their RNA into complementary DNA (cDNA), amplifying the cDNA, and then sequencing it to quantify gene expression. Various platforms, such as droplet-based methods (10x Genomics) and plate-based methods (SMART-Seq), have been developed, each with specific advantages in terms of throughput and sensitivity (16). These advancements have made it possible to profile thousands to millions of cells in a single experiment, greatly enhancing our understanding of cellular diversity (38).

scRNA-seq technologies have become pivotal in advancing our understanding of Alzheimer’s disease (AD), enabling detailed analyses of cellular heterogeneity and molecular mechanisms. Two of the most widely used platforms for scRNA-seq are droplet-based methods (e.g., 10x Genomics, Drop-seq) and plate-based methods (e.g., SMART-seq, CEL-Seq), each with distinct strengths and limitations (39). Droplet-based methods, such as 10x Genomics, are known for their high throughput, enabling the processing of thousands to millions of cells, which is particularly beneficial for large-scale studies of AD pathology. However, their sensitivity may be lower, especially when analyzing rare cell types, as these methods can miss low-abundance populations (15, 40). In contrast, plate-based methods like SMART-seq offer higher sensitivity and better detection of rare transcripts, making them ideal for studying specific cell types like neurons or microglia in AD, which are crucial for understanding disease progression. These methods provide more detailed transcriptomic profiles but come with higher costs and lower throughput compared to droplet-based methods, making them more suited for focused studies on particular cell populations (41). A comparison of the two platforms highlights their respective advantages: droplet-based methods are more scalable and cost-effective, while plate-based methods excel in capturing more nuanced and rare transcriptomic information, crucial for dissecting the molecular basis of AD (42). By selecting the appropriate platform, researchers can tailor their approach to the specific needs of their AD studies, balancing between scale and resolution.

The application of single-cell transcriptomics to AD research has evolved significantly since its inception. Initial studies focused on bulk RNA sequencing, which, while informative, masked the contributions of individual cell types to disease pathology (43). The first major scRNA-seq study in AD was conducted by Mathys et al. (15), who profiled approximately 80,000 cells from the prefrontal cortex of individuals with varying degrees of AD pathology, identifying cell-type-specific transcriptional changes and revealing the complexity of the disease at a cellular level. Since then, numerous studies have expanded on this work, utilizing advanced single-cell and single-nucleus RNA sequencing techniques to dissect the transcriptomic landscapes of different brain regions and cell types in AD (34). These studies have uncovered novel insights into the roles of specific cell types, such as microglia and oligodendrocytes, in disease progression, and have identified key molecular pathways involved in AD (18).

Single-cell transcriptomics has fundamentally transformed our understanding of Alzheimer’s disease by highlighting the cellular heterogeneity within affected brain regions. It has revealed distinct transcriptional profiles of neurons, glial cells, and other brain cell types, showing how these profiles change with disease progression (15, 41). For instance, scRNA-seq studies have identified disease-associated microglial subpopulations that exhibit upregulated inflammatory responses, suggesting a critical role for these cells in AD pathology (16). Moreover, single-cell transcriptomics has facilitated the identification of cell-type-specific gene expression changes associated with AD risk genes, providing deeper insights into the molecular mechanisms underlying the disease (16). This technology continues to drive forward our understanding of AD, paving the way for the development of targeted therapeutic strategies (37).

### 3.2 Cellular heterogeneity in Alzheimer’s disease by single-cell transcriptomics

Significant findings from single-cell transcriptomics studies in AD have underscored the complex and multifaceted nature of the disease. Mathys et al. (15) identified transcriptionally distinct subpopulations across six major brain cell types, including those associated with AD pathology. This study revealed that early disease-associated changes are highly cell-type specific, whereas later changes involve global stress responses common across cell types. Another major discovery by Grubman et al. (16) highlighted the cell-type-specific gene expression regulation in the entorhinal cortex of AD patients. They found that the Alzheimer’s disease risk gene APOE is differentially regulated across various cell types, suggesting diverse roles in disease progression. Furthermore, a study by Morabito et al. (43) utilized a multi-omic approach to identify disease-associated regulatory elements and target genes, providing insights into the gene-regulatory mechanisms in AD.

These discoveries have significantly impacted our understanding of Alzheimer’s disease pathogenesis. The identification of cell-type-specific gene expression changes has revealed how different brain cells contribute to AD in unique ways (15). For example, the discovery of disease-associated microglial subpopulations has highlighted the importance of neuroinflammation in AD (16). Similarly, the identification of oligodendrocyte-associated regulatory modules linked to AD risk genes has emphasized the role of myelination in disease progression (43). These insights have also provided new avenues for therapeutic intervention. By targeting specific cell types and their associated pathways, it may be possible to develop more effective treatments for AD (41). The understanding of transcriptional changes at a single-cell level allows for the precise modulation of disease-relevant pathways, potentially leading to personalized medicine approaches for AD.

### 3.3 Single-cell insights into disease-associated microglia

scRNA-seq has unveiled the critical roles of disease-associated microglia (DAM) in Alzheimer’s disease (AD), providing novel insights into its pathogenesis. DAM exhibits a stratified activation pattern with early and late states. Early DAM activation occurs through a TREM2-independent pathway, characterized by the upregulation of genes associated with phagocytic function, such as APOE and CSF1R, enabling the clearance of Aβ plaques. In contrast, late-stage DAM depends on TREM2 signaling and exhibits increased expression of pro-inflammatory genes, such as IL-1β and TNF-*α*, leading to chronic neuroinflammation and neuronal damage. These dual roles highlight DAM’s protective function in early-stage AD while contributing to exacerbated pathology in later stages (17, 44, 45).

Gene network analyses have provided a deeper understanding of DAM stratification mechanisms. Early DAM is regulated by key genes such as TREM2, APOE, and LPL, which are critical in lipid metabolism and phagocytic pathways. Transcription factors, including SPI1 and MEF2, have been identified as central regulators in DAM activation and AD risk modulation (15, 46, 47). Single-cell studies have revealed the dynamic transcriptional changes in DAM across AD stages, emphasizing their potential as biomarkers and therapeutic targets.

The discovery of DAM has significant clinical implications. DAM states are potential biomarkers for monitoring early AD progression and neuroinflammation. Moreover, therapeutic strategies targeting DAM, such as modulating TREM2 signaling to enhance Aβ clearance or inhibiting late DAM’s pro-inflammatory cytokines, hold promise for mitigating AD progression (48, 49). Future research should focus on elucidating DAM’s regulatory mechanisms and exploring species-specific differences to advance precision medicine approaches in AD treatment.

## 4 Spatial transcriptomics in Alzheimer’s disease

### 4.1 Spatial distribution of immune cells

Spatial transcriptomics, utilizing techniques such as *in situ* hybridization and spatially resolved RNA-seq, has revolutionized our understanding of the spatial distribution of immune cells in AD (19). These methodologies enable the detailed mapping of gene expression across different brain regions, providing insights into the localization and density of immune cells in both healthy and AD-affected brains (14). For instance, spatial transcriptomic analysis using the 10x Genomics Visium platform has revealed significant changes in the distribution patterns of immune cells in various brain regions such as the hippocampus and cortex in AD models compared to healthy controls (20). The Visium platform combines spatially resolved transcriptomic data with histological images, enabling researchers to link gene expression patterns directly to tissue morphology. This technology has been particularly effective in mapping amyloid-beta (Aβ) and tau pathology in brain regions affected by AD, revealing spatially distinct gene expression signatures associated with disease progression (16). By contrast, MERFISH uses highly multiplexed *in situ* hybridization to achieve single-molecule resolution, allowing for the identification and localization of hundreds to thousands of genes simultaneously. This capability is especially valuable for dissecting cell–cell interactions in AD, such as the interplay between microglia and astrocytes in response to neuroinflammation (50).

In AD brains, there is a marked alteration in the spatial distribution of immune cells, including microglia and astrocytes, which are closely associated with amyloid plaques and neurofibrillary tangles. Studies have demonstrated increased microglial activation and clustering around amyloid plaques, suggesting a role in plaque clearance and neuroinflammation (19, 51). Comparisons between healthy and AD brains indicate not only changes in cell density but also significant shifts in the activation states of these immune cells, which may contribute to the progression of neurodegenerative pathology (52).

### 4.2 Neuroimmune interactions

The interplay between neurons and immune cells in AD involves complex mechanisms such as synaptic pruning and neuroinflammation. Spatial transcriptomics has uncovered critical insights into how these interactions exacerbate neuronal dysfunction and cell death (53). For instance, microglia-mediated synaptic pruning, which is essential for normal brain development, becomes dysregulated in AD, leading to excessive synapse loss and cognitive decline (19).

Neuroimmune interactions also significantly impact AD pathology through mechanisms involving the complement system, oxidative stress, and lysosomal pathways. These interactions are spatially and temporally dynamic, influencing the progression of AD by affecting neuronal health and synaptic integrity (54). Specific case studies have highlighted how spatial transcriptomics can map the interactions between neurons and immune cells at high resolution, providing a detailed analysis of key molecular pathways involved in AD (19, 55).

### 4.3 Cellular spatial patterns in Alzheimer’s disease

Disease-associated microglia (DAM) are predominantly localized around amyloid-beta (Aβ) plaques, where they are involved in early Aβ clearance but also contribute to neuroinflammation in late stages. Activated astrocytes are distributed densely in the prefrontal cortex and hippocampus, where they release ATP to activate P2X7 receptors, triggering inflammatory cascades. Moreover, pathological neurons displaying Tau protein hyperphosphorylation and synaptic loss are primarily observed in the hippocampal CA1 region, correlating with early cognitive deficits (19, 54).

The hippocampus, a region critical for memory, exhibits significant early-stage pathological changes, such as Tau phosphorylation and synaptic degeneration. In contrast, the prefrontal cortex demonstrates broader inflammatory activation, particularly in later stages of AD. Spatial transcriptomics has enabled detailed mapping of these region-specific cellular and molecular alterations, revealing distinct microglial, astrocytic, and neuronal responses across brain regions. This regional heterogeneity underscores the complexity of AD pathogenesis and highlights the importance of spatially resolved data to link cellular dysfunction to pathology (50, 53).

Spatial transcriptomics has further advanced the study of gene expression dynamics near Aβ plaques (19). High-resolution techniques, such as Stereo-seq and CosMx, have revealed coordinated microglial-astrocytic responses, complement activity, oxidative stress, and inflammation in the plaque microenvironment (55). These findings suggest a progression from Aβ-driven to inflammation-driven pathology as the disease advances. By integrating spatial transcriptomics with advanced computational approaches, researchers are now able to dissect the molecular and cellular networks driving AD pathogenesis, providing new targets for therapeutic intervention (53).

## 5 Novel neuroimmune markers in Alzheimer’s disease

### 5.1 Identification of biomarkers

Recent advancements in methods such as single-cell RNA sequencing (scRNA-seq), spatial transcriptomics, and proteomics have significantly enhanced our ability to identify novel biomarkers for AD. Single-cell RNA-seq allows for the dissection of complex tissues into individual cell types, revealing distinct molecular signatures associated with AD (56). Spatial transcriptomics adds another layer by preserving the spatial context of gene expression within the brain, which is crucial for understanding the microenvironmental influences on AD pathology (54). Proteomics, particularly through the analysis of brain-derived exosomal proteins, has identified potential biomarkers such as amyloid-*β*42, total tau, and phosphorylated tau (56).

Key biomarkers identified through these techniques include specific proteins and RNAs that play pivotal roles in the pathogenesis of AD. For instance, amyloid-β and tau proteins are well-established markers, but recent studies have highlighted the importance of other molecules such as neurofilament light (NfL) and phosphorylated tau181 (p-tau181), which have shown promise in distinguishing AD patients from healthy controls (57). These biomarkers not only aid in early diagnosis but also hold potential for predicting disease progression and response to treatment (58).

### 5.2 New biomarkers in Alzheimer’s disease: p-Tau181 and NfL

Alzheimer’s disease (AD) is characterized by the accumulation of amyloid-beta (Aβ) plaques and tau neurofibrillary tangles, leading to neurodegeneration and cognitive decline. Recent advancements in biomarker research have highlighted the significance of phosphorylated tau at threonine 181 (p-tau181) and neurofilament light chain (NfL) as promising indicators for diagnosing and monitoring the progression of AD.

p-Tau181 has emerged as a critical biomarker due to its strong association with tau pathology in the brain. Studies have demonstrated that elevated levels of plasma p-tau181 correlate with the presence of amyloid pathology and tau deposition as measured by positron emission tomography (PET) scans. In particular, p-tau181 has shown high diagnostic accuracy in distinguishing AD from other neurodegenerative disorders, with area under the curve (AUC) values indicating excellent performance (59). Furthermore, longitudinal studies suggest that p-tau181 levels can predict cognitive decline and neurodegeneration, making it a valuable tool for early detection and monitoring of disease progression (60). Neurofilament light chain (NfL) is another emerging biomarker that reflects axonal damage and neurodegeneration. Elevated NfL levels in both cerebrospinal fluid (CSF) and plasma have been associated with various neurodegenerative diseases, including AD (27, 61). Research indicates that NfL levels correlate with cognitive impairment and can serve as a prognostic marker for disease progression (10, 44). The combination of NfL with other biomarkers, such as p-tau181, enhances diagnostic accuracy and provides a more comprehensive understanding of the underlying pathology in AD (27, 61).

The integration of p-tau181 and NfL into clinical practice could revolutionize the approach to diagnosing and managing Alzheimer’s disease. These biomarkers not only facilitate early detection but also offer insights into the disease’s trajectory, enabling personalized treatment strategies. As research continues to validate these biomarkers, they hold the potential to significantly improve outcomes for individuals at risk for or diagnosed with Alzheimer’s disease.

### 5.3 Comparative analysis of Alzheimer’s disease and other neurodegenerative diseases

Alzheimer’s disease (AD) is the most prevalent form of neurodegenerative disorders, characterized by progressive cognitive decline and specific neuropathological features such as amyloid-beta plaques and neurofibrillary tangles. However, it is essential to understand how AD compares and contrasts with other neurodegenerative diseases, such as Parkinson’s disease (PD), frontotemporal dementia (FTD), and dementia with Lewy bodies (DLB). Recent studies have highlighted commonalities in biological pathways, genetics, and cellular mechanisms between AD and other neurodegenerative diseases. For instance, both AD and PD share similar inflammatory responses and oxidative stress pathways that contribute to neuronal degeneration (62, 63). Additionally, the role of tau protein in AD is mirrored by its involvement in other tauopathies, suggesting a shared mechanism of neurodegeneration across these diseases (64). Moreover, the differential diagnosis of AD from other neurodegenerative disorders remains a challenge due to overlapping symptoms. For example, olfactory dysfunction has been identified as a potential early marker for AD, but similar deficits are also observed in PD and DLB. This indicates that while there are distinct pathological features associated with each disease, there are also significant overlaps that complicate clinical assessments.

The use of biomarkers has become increasingly important in differentiating AD from other neurodegenerative diseases. For instance, cerebrospinal fluid (CSF) levels of specific proteins, such as *α*-synuclein, have been shown to vary significantly between AD and PD, providing a potential avenue for accurate diagnosis (65). Additionally, recent advancements in epigenetic profiling have revealed distinct signatures that may help in identifying specific neurodegenerative conditions, including AD (66). Furthermore, lifestyle factors, such as physical exercise, have been shown to influence the progression of AD and other neurodegenerative diseases (67). Long-term treadmill exercise has been reported to reduce amyloid-beta burdens and improve cognitive function in animal models of AD. This suggests that interventions targeting lifestyle modifications could have broader implications for managing various neurodegenerative diseases. While Alzheimer’s disease is a distinct entity within the spectrum of neurodegenerative disorders, its pathophysiology shares common features with other diseases. Understanding these similarities and differences is crucial for developing effective diagnostic and therapeutic strategies.

### 5.4 Validation and functional studies

The validation of these biomarkers is crucial for their adoption in clinical settings. Techniques such as immunohistochemistry and flow cytometry are commonly used for this purpose. Immunohistochemistry allows for the visualization of biomarker distribution within brain tissues, confirming their presence and relevance in AD pathology (68). Flow cytometry, on the other hand, enables the quantification of biomarkers in blood samples, providing a less invasive method for monitoring disease progression (69).

Functional studies further elucidate the roles of these biomarkers in AD. *In vitro* and *in vivo* models, including the use of CRISPR/Cas9 for gene editing, have been instrumental in understanding how specific biomarkers contribute to disease mechanisms. For instance, CRISPR/Cas9 has been used to manipulate genes associated with amyloid-*β* production, providing insights into the molecular pathways involved in plaque formation (70). *In vivo* studies using transgenic mouse models have demonstrated how alterations in biomarkers such as p-tau181 can influence cognitive decline and neurodegeneration (71).

Case studies provide concrete examples of validated biomarkers and their functional roles in AD pathology. For example, plasma p-tau181 has been shown to predict cognitive decline and hippocampal atrophy in patients, underscoring its utility as a prognostic marker (32). Similarly, the combination of multiple biomarkers, such as amyloid-β42/40 ratio, NfL, and p-tau181, has been effective in predicting disease progression in cognitively unimpaired elderly individuals (69).

## 6 Therapeutic implications

### 6.1 Targeting neuroimmune pathways

Current therapeutic strategies for AD increasingly focus on modulating neuroimmune pathways. Anti-inflammatory drugs, immunomodulators, and antibody therapies are among the most prominent approaches. For instance, monoclonal antibodies such as lecanemab and aducanumab target amyloid-*β* (Aβ) to reduce its aggregation, thereby addressing one of the primary pathological features of AD (72). Anti-inflammatory drugs aim to mitigate the chronic neuroinflammation seen in AD by targeting pathways like the NF-κB and NLRP3 inflammasome pathways, which are crucial in mediating inflammatory responses in the brain (73).

Recent advancements in single-cell sequencing and spatial transcriptomics have provided new insights into potential therapeutic targets. These techniques have identified specific immune cell subsets and signaling pathways that are dysregulated in AD. For example, studies have highlighted the role of microglia and their associated pathways, such as the CSF1R signaling, as critical regulators of neuroinflammation and potential therapeutic targets (74). By modulating these pathways, therapies can potentially reduce neuroinflammation and promote neuroprotection, thereby slowing the progression of AD (75).

**Figure 3:**
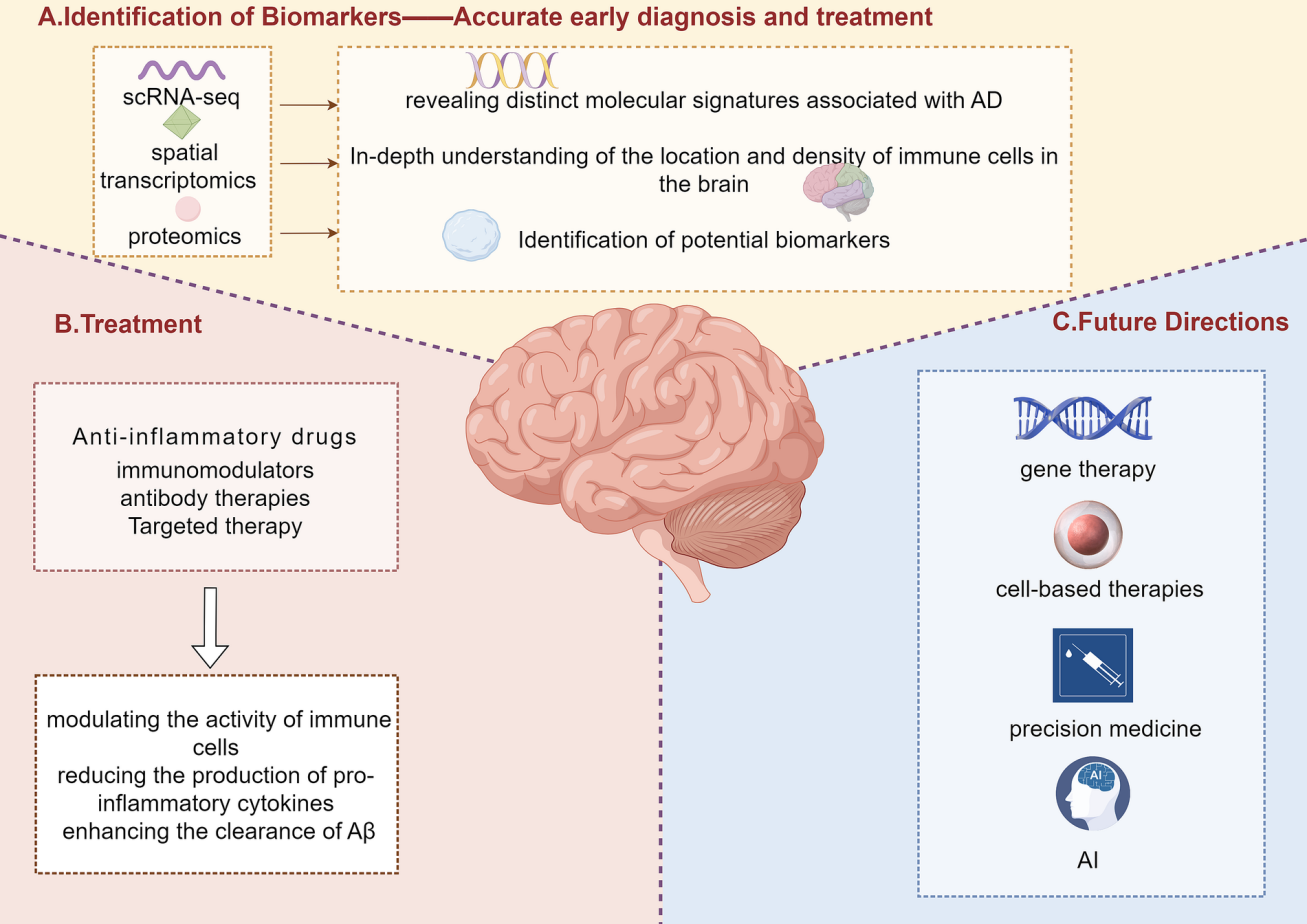
Identification of biomarkers, treatment strategies, and future directions in Alzheimer’s disease. This figure illustrates a comprehensive approach to Alzheimer’s disease (AD) research, including the identification of biomarkers, treatment strategies, and future directions. Panel A highlights the unique advantages of single-cell RNA sequencing (scRNA-seq), spatial transcriptomics, and proteomics in identifying novel biomarkers for AD. These technologies can identify specific proteins and RNAs that play a key role in the pathogenesis of AD, providing more accurate early diagnosis and more effective treatment. Panel B outlines current treatment strategies, including anti-inflammatory drugs, immunomodulators, antibody therapies, and targeted therapies, which involve modulating immune cell activity, reducing the production of pro-inflammatory cytokines, and enhancing the clearance of amyloid-beta (Aβ). Panel C presents future directions in AD treatment, including gene therapy, cell-based therapies, precision medicine, and artificial intelligence (AI) applications.

The mechanisms of action of these therapies often involve modulating the activity of immune cells, reducing the production of pro-inflammatory cytokines, and enhancing the clearance of Aβ. For instance, anti-inflammatory drugs can inhibit the activation of microglia and astrocytes, reducing the release of neurotoxic substances and mitigating neuronal damage (76). Immunomodulators and antibody therapies work by binding to specific targets such as Aβ or tau proteins, preventing their aggregation and facilitating their clearance from the brain (77) (Figure 3).

### 6.2 Future directions

Emerging therapies for AD include gene therapy, cell-based therapies, and precision medicine approaches. Gene therapy aims to correct genetic mutations associated with AD or to enhance the expression of protective genes. Techniques such as CRISPR/Cas9 allow for precise genetic modifications, offering potential cures by targeting the underlying genetic causes of AD (70). Cell-based therapies, including the use of stem cells, aim to replace damaged neurons and support neuroregeneration, which could significantly improve cognitive functions in AD patients (61).

Challenges and opportunities in the development of these therapies include translational hurdles, the need for personalized medicine approaches, and ethical considerations. Translating findings from animal models to human patients is often challenging due to differences in physiology and disease progression (78). Personalized medicine approaches, which tailor treatments based on an individual’s genetic profile, can enhance the efficacy of therapies but require extensive genetic and biomarker profiling (79). Ethical considerations, such as the long-term effects and potential risks of gene editing and stem cell therapies, must also be addressed to ensure patient safety and public acceptance (80).

Research gaps that need further investigation include understanding the complex interactions between different pathological pathways in AD and identifying reliable biomarkers for early diagnosis and treatment monitoring. Studies are needed to explore the roles of neuroinflammation, oxidative stress, and other pathological mechanisms in greater detail to develop more effective multi-target therapies (81). Potential breakthroughs in these areas could lead to the development of novel therapies that not only alleviate symptoms but also modify the disease course, offering hope for a cure for AD.

## 7 Integration of big data and AI in neuroimmune research

### 7.1 Role of big data in single-cell sequencing

The integration of big data in single-cell sequencing has transformed our ability to understand the complex neuroimmune interactions in Alzheimer’s disease (AD). Techniques for handling large datasets, such as data harmonization and advanced computational algorithms, are essential for processing the vast amounts of information generated by scRNA-seq and spatial transcriptomics (20). These methods enable researchers to combine data from multiple studies, enhancing the robustness and reproducibility of findings (18).

Big data contributions have provided comprehensive views of neuroimmune interactions, uncovering new discoveries about cellular and molecular mechanisms in AD. For instance, the integration of large-scale single-cell datasets has revealed previously unrecognized subpopulations of immune cells and their roles in AD pathology (25). By analyzing the gene expression profiles of individual cells, researchers can identify specific markers and pathways involved in neuroinflammation and neurodegeneration, leading to a deeper understanding of disease progression (21).

Case studies have demonstrated the power of big data applications in AD research. The ssREAD database, for example, compiles over 189 datasets from single-cell and spatial transcriptomics studies, providing a valuable resource for the scientific community (41). This database allows researchers to perform integrative analyses, such as cell clustering and identification of differentially expressed genes, facilitating the discovery of new therapeutic targets and biomarkers for AD (20).

### 7.2 AI and machine learning applications

Artificial intelligence (AI) and machine learning (ML) applications have significantly enhanced data analysis in neuroimmune research. Machine learning algorithms and AI-based predictive models enable the extraction of meaningful patterns from complex datasets, improving the accuracy of disease modeling and prediction of disease progression (42). These technologies allow for the integration of multi-omics data, combining genetic, proteomic, and clinical information to provide a holistic view of AD (82).

In research applications, AI has been used to identify therapeutic targets by analyzing single-cell sequencing data. For instance, AI-driven analyses have pinpointed specific immune cell types and signaling pathways that are dysregulated in AD, offering new avenues for therapeutic intervention (31). Additionally, machine learning models have been employed to predict patient responses to treatments, paving the way for personalized medicine approaches in AD care (83).

Future prospects for AI in neuroimmune research include AI-driven drug discovery and integration with clinical practice. AI algorithms can screen vast libraries of compounds to identify potential drugs that modulate neuroimmune pathways, accelerating the drug development process (84). Moreover, integrating AI tools into clinical practice can enhance diagnostic accuracy and treatment efficacy, providing personalized care based on an individual’s genetic and molecular profile (83). However, challenges such as data privacy, algorithm transparency, and the need for large, high-quality datasets must be addressed to fully realize the potential of AI in this field (85).

## 8 Conclusion

Recent advancements in single-cell sequencing and spatial transcriptomics have significantly enhanced our understanding of AD. These technologies have revealed the complexity and heterogeneity of brain cell types, identifying distinct cellular subpopulations and specific gene expression profiles associated with AD. Key findings include the discovery of disease-associated microglial subpopulations and cell-type-specific regulatory elements linked to AD risk genes, which provide new insights into the molecular mechanisms underlying the disease and potential therapeutic targets (15, 16, 19, 43).

Looking forward, single-cell and spatial transcriptomics are poised to drive new research paradigms and personalized therapeutic approaches for AD. By continuing to integrate these technologies with other omics data, researchers can develop a more comprehensive understanding of the disease, ultimately leading to the identification of novel therapeutic strategies. Collaboration, funding, and interdisciplinary efforts will be essential to advance this field and improve patient outcomes (20).

## Glossary

AD: Alzheimer’s Disease
MCI: Mild Cognitive Impairment
Aβ: Amyloid-beta
scRNA-seq: Single-Cell RNA Sequencing
NF-κB: Nuclear Factor Kappa-light-chain-enhancer of Activated B cells
NLRP3: NACHT, LRR, and PYD domains-containing protein 3
IL-1β: Interleukin-1 beta
DAM: Disease-Associated Microglia
APOE: Apolipoprotein E
NfL: Neurofilament Light
p-tau181: Phosphorylated Tau 181
CRISPR/Cas9: Clustered Regularly Interspaced Short Palindromic Repeats/Cas9
AI: Artificial Intelligence
ML: Machine Learning
RNA: Ribonucleic Acid
cDNA: Complementary DNA
ATP: Adenosine Triphosphate
CSF1R: Colony Stimulating Factor 1 Receptor
TACA: The Alzheimer’s Cell Atlas
RNA-seq: RNA Sequencing
ssREAD: Single-Cell RNA Expression Analysis Database
HER2: Human Epidermal Growth Factor Receptor 2
LPS: Lipopolysaccharide
Nrf2: Nuclear factor erythroid 2-related factor 2
TNF: Tumor Necrosis Factor
IL2: Interleukin 2
